# Comparative analysis of the relationship between four hepatic steatosis indices and muscle mass

**DOI:** 10.1038/s41598-023-28751-5

**Published:** 2023-01-30

**Authors:** Taesic Lee, Tae-Ha Chung

**Affiliations:** 1 Department of Family Medicine, Yonsei University Wonju College of Medicine, 20 Ilsan-ro, Wonju, 26426 Republic of Korea; 2 The Study of Obesity and Metabolic Syndrome, Korean Academy of Family Medicine, Wonju, Republic of Korea; 3 Research Group of Functional Medicine and Preclinical Disease, Wonju, Republic of Korea

**Keywords:** Endocrine system and metabolic diseases, Metabolic disorders

## Abstract

Several studies have attempted to validate the relationship between hepatic steatosis and sarcopenia. The crucial limitation is to establish the status of hepatic steatosis by costly or invasive methods. Therefore, several models predicting non-alcoholic fatty liver disease (NAFLD) have been developed but have exhibited heterogeneous results. In this study, we aimed to review and compare four representative models and analyze their relationship with the risk of low muscle mass. Korea National Health and Nutrition Examination Surveys from 2008 to 2011 were used to confirm our hypothesis. Dual-energy X-ray absorptiometry was used to measure the amount of skeletal muscle mass. We used four hepatic steatosis indices: hepatic steatosis index (HSI), Framingham steatosis index (FSI), liver fat score (LFS), and fatty liver index (FLI). Multivariate linear and logistic regressions were used to reveal the relationship between NAFLD and low skeletal muscle index (LSMI). Pairs of FSI-FLI and HSI-FLI exhibited the best and second-best correlations among all possible pairs. The four hepatic steatosis models were associated with increased risk for LSMI. After removing the body mass index effect, HSI and FLI remained robust predictors for LSMI. NAFLD was a significant and potent risk factor for low skeletal muscle.

## Introduction

Sarcopenia is defined as a progressive and generalized muscle disorder related to increased risk for adverse outcomes such as falls, fractures, physical disability, and mortality^1,2^. In 2010, the European Working Group on Sarcopenia in Older People (EWGSOP) defined sarcopenia by focusing on low muscle mass, specifically skeletal muscle^1^. In 2018, the group of experts met again (EWGSOP2) to provide a revised definition, which included more scientific and biological evidence and stipulated muscle strength as additional diagnostic component for sarcopenia^2^. Decreased muscle mass or strength could develop mainly with aging, which is called age-related sarcopenia or primary sarcopenia^1–4^. Moreover, sarcopenia can be caused by chronic disorders such as metabolic disease, cancer, chronic kidney disease, chronic obstructive pulmonary disease, and neuromuscular disease^1–4^ and is called secondary sarcopenia.

In 2014, Hong et al.^5^ demonstrated that low skeletal muscle index (LSMI) was significantly related to an increased risk of predicting non-alcoholic fatty liver disease (NAFLD) diagnosed using unenhanced computed tomography (CT). Later, in 2015, Lee et al.^6^ reported that sarcopenia exhibited a significant association with NAFLD independent of obesity or insulin resistance. Recently, Kang et al.^7^ showed that weak muscle strength was related to increased prevalence of NAFLD as well as its severity based on several indices estimating hepatic steatosis and fibrosis.

Radiological findings or histological confirmation is required to determine the status of hepatic steatosis. However, these modalities are costly and invasive procedures. Therefore, several prediction models for hepatic steatosis have been developed^8–11^. Recently, comparative studies were conducted to validate predictive models^12,13^. This study reviewed and compared four representative models for predicting hepatic steatosis. Afterward, we analyzed the relationships of each model with LSMI and compared their four-pair relational trends. Body mass index (BMI) is included as a predictor for most NAFLD prediction models as well as a diagnostic marker of sarcopenia. Therefore, we compared the change of relational direction between LSMI and each NAFLD model before and after removing the effect of BMI.

## Methods

### Study population

We analyzed a cross-sectional study using the 2008–2011 Korea National Health and Nutrition Examination Surveys (KNHANES). KNHANES is a nationwide, representative, and population-based survey conducted annually by the Korean Centers for Disease Control and Prevention (K-CDC). Detailed information about these data has been reported^14,15^. Subjects aged < 20 years were excluded, and we conducted the following steps to select the final samples.

Step (1) We estimated the average amount of alcohol consumption per week using questionnaire-based information (covariates section). Lee et al.^6^ excluded men and women with alcohol consumption >140 g/week and >70 g/week, respectively. In another study by Kang et al.^7^, subjects who consumed >210 g/week (men) and >140 g/week (women) were eliminated to analyze NAFLD. Lee et al.^16^ excluded men and women who consume ≥30 g/day and ≥20 g/day of alcohol, respectively. Among these criteria, we selected the gender-specific cut-offs for categorizing the alcohol group determined by Lee et al.^6^ and excluded non-alcoholic subjects who consumed more than 140 g/week and 70 g/week in men and women, respectively.

Step (2) Several studies have conducted analyses after excluding subjects with liver disease. Lee et al.^6^ excluded subjects with evidence of hepatitis B or C virus and had previously been diagnosed to have liver cirrhosis. In addition, several studies eliminated positive hepatitis B antigens or hepatitis C antibodies^7,16,17^. In our study, any subject having positive serologic markers for viral hepatitis and liver cirrhosis diagnosis were excluded.

Step (3) Lee et al.^6^ retained subjects if any of the four hepatic steatosis indices could be calculated. In another study, subjects whose hepatic steatosis index could not be calculated due to missing values of clinical and laboratory variables were excluded^16^. In the present study, we only excluded subjects without any indices for hepatic steatosis.

Step (4) Subjects who did not have a measurement of body composition were excluded.

Finally, we selected 12,324 subjects (men: 4201, women: 8123) to identify the association between muscle mass and NAFLD. Note that subjects only enrolled in the KNHANES were used to obtain the Korean population's generalized findings.

The present study was approved by the Institutional Review Board (IRB) of the K-CDC (IRB number: 2008-04EXP-01-C, 2009-01CON-03-2C, 2010-02CON-21-C, 2011-02CON-06-C). The present study was performed in accordance with the Declaration of Helsinki.

### Measurement of body composition and muscle mass

Dual-energy X-ray absorptiometry (DEXA) was used to measure the body composition data collected for the head, trunk, pelvis, arms, legs, and whole body. Skeletal muscle mass (SMM) was calculated by subtracting the lean body mass (g) from bone mineral content (g). The appendicular skeletal muscle mass (ASM) was measured by the sum of SMM for the two arms and two legs^1,2^. The skeletal muscle index (SMI) was calculated by dividing ASM by BMI. Based on the Foundation for the National Institutes of Health (FNIH) Sarcopenia Project criteria^18^, gender-specific LSMI was defined as SMI < 0.789 in men and < 0.512 in women.

### Calculation of indices for NAFLD

Numerous models for predicting NAFLD have been established and validated for their clinical usefulness in predicting CVD and cancers^19,20^. The KNHANES that we analyzed includes incomplete clinical and laboratory data for calculating certain NAFLD models. For example, because the KNHANES does not compose serum uric acid, it cannot calculate the Comprehensive NAFLD score^21^. Therefore, we used four indices for screening NAFLD that are computable from the KNHANES: the hepatic steatosis index (HSI)^8^, Framingham steatosis index (FSI)^9^, liver fat score (LFS)^10^, and fatty liver index (FLI)^11^.

The HSI was based on 10,724 subjects (derivation set, 5360; validation set, 5364) who visited Seoul National University Hospital Gangnam Healthcare Center^8^. Multivariate logistic regression was used to select risk factors for NAFLD, and the model for predicting NAFLD was constructed with training parameters using logistic regression, scaling optimized parameters, and adjusting parameters for gender. The HSI used ultrasonography (US) examinations as the actual label for NAFLD.

The FSI was established using 1181 participants in the Framingham Heart Study Third Generation Cohort. Clinical and laboratory biomarkers were determined by integrating expert knowledge (i.e., gender) and stepwise logistic regression. The model for predicting hepatic steatosis was constructed using logistic regression. A multi-detector CT scan was used to measure the level of liver attenuation^9^.

The LFS was based on Finnish subjects in which the ratio of type 2 diabetes is 0.23 (111/470)^10^. Predictors for the LFS were selected using multivariate backward stepwise logistic regression, and the prediction model for NAFLD was established using logistic regression. The liver fat content was measured using proton magnetic resonance spectroscopy.

The FLI had been curated using 496 Italian subjects (216 subjects with and 280 without suspected liver disease) enrolled in the Dionysos Nutrition & Liver Study^11^. By integrating bootstrap and stepwise logistic regression, potential predictors were selected, and the model for predicting fatty liver was constructed using logistic regression. This study used US examinations to diagnose NAFLD.

### Covariates

We reviewed about 20 covariates previously used for multivariate logistic or Cox proportional hazard regression models in four studies^6,7,16,17^ (Table 1). Among them, we did not include C-reactive protein, homeostatic model assessment for insulin resistance (HOMA-IR), or serum levels of vitamin D, hemoglobin A1c (HbA1c), and uric acid as covariates in our study because these values were missing in about half of the subjects in KNHANES 2008–2011. Race and ethnicity were not included in this study because only Koreans were analyzed in the dataset.

**Table 1:** Covariates used for the multivariate model in previous studies.

Covariates	Lee et al. (2015)^6^	Peng et al. (2019)^17^	Kang et al. (2020)^7^	Lee et al. (2021)^16^
Age	O	O	O	O
Sex	O	O	O	O
Race/ethnicity		O		
Physical activity/ Regular exercise	O	O		O
Smoking status	O	O		O
Drinking status				O
Daily protein intake				O
Hypertension	O		O	
Diabetes mellitus			O	
Dyslipidemia			O	
CVD				O
Obesity			O	O
MAP				O
Serum fasting glucose				O
Total cholesterol		O		O
C-reactive protein		O	O	
HOMA-IR	O		O	
Serum vitamin D level		O		
Serum HbA1c		O		
Serum uric acid		O		

*CVD* cardiovascular disease, *MAP* mean arterial pressure, *HOMA-IR* homeostatic model assessment for insulin resistance.

The presence of physical activity (PA) or regular exercise was defined by Lee et al.^6^ as vigorous exercise ≥ 20 min at a time and ≥ 3 times per week. Peng et al.^17^ determined PA as 3 to 6 metabolic equivalents (METs) ≥ 5 times per week or more than six METs ≥ 3 times per week. Lee et al.^16^ defined vigorous exercise as ≥ 20 min at least three days per week and moderate exercise as ≥ 30 min (or walking) at least 5 days per week. We determined the presence of regular exercise when a subject satisfied one or more of the above criteria^6,16,17^.

Smoking status is typically categorized into three groups: non-, ex-, and current smokers^6^. Peng et al.^17^ defined a person who smokes at least 100 cigarettes during his or her lifetime as a smoker. In the eminent atherosclerotic cardiovascular disease (ASCVD) models^22–24^, smoking status is considered one of the crucial predictors for ASCVD and is implicated as the binary form of current smoking or not. The present study used the binary form of smoking (current vs. ex- and non-smoker) as a covariate.

For the definition of drinking status, we measured the total amounts of alcohol consumption in each subject, similar to the study by Lee et al.^6^. However, several studies initially excluded subjects with high alcohol consumption and did not include alcohol consumption as a covariate^6,7,17^. Therefore, we did not consider it. For daily protein intake (g/day), the 24-h recall method was conducted by well-trained dietitians. Nutritional contents, such as daily intakes of carbohydrates and protein, were measured using the Korean Food Composition Table provided by the Rural Development Administration of Korea^25^.

We defined subjects with hypertension based on the Seventh Report of the Joint National Committee as follows: systolic blood pressure (BP) ≥ 140 or diastolic BP ≥ 90 mmHg; previous diagnosis of hypertension by a medical doctor; and antihypertensive medications^26^. Diabetes mellitus was defined as serum fasting glucose level ≥ 126 mg/dL; previous diagnosis by a medical doctor; or glucose-lowering drugs. Kang et al.^7^ used both questionnaire- and laboratory-based information for the status of dyslipidemia. However, too many subjects missing data on serum level of high-density lipoprotein (HDL) cholesterol. Therefore, we only depended on the questionnaire-based information, including previous diagnoses by a doctor and anti-lipidemic drug administration to determine the status of dyslipidemia.

Several studies used obesity status as a covariate^7,16^. One study used obesity defined by BMI^7^, and another determined obesity by waist circumference (WC)^16^. However, because three of the four models for predicting fatty liver included BMI as a predictor (Table 2), the BMI-based definition of LSMI was strongly predicted to be significantly related to the BMI-based fatty liver indices. Therefore, we did not use obesity as a covariate. Serum creatinine level is known to be significantly associated with muscle mass^27^. Thereby, it was included as a confounder.

**Table 2:** Features and their coefficients in the NAFLD scoring models.

Features	HSI	FSI	LFS	FLI
Age	–	0.011	–	–
Female	2; score^a^	–0.146	–	–
Body mass index, kg/m^2^	1.0	0.173	–	0.139
Waist circumference, cm	–	–	–	0.053
Metabolic syndrome	–	–	1.18	–
Hypertension	–	0.593	–	–
Type 2 diabetes	2; score^a^	0.789	0.45	–
AST, U/L	–	–	0.04	–
AST/ALT ratio	–	–	–0.94	–
ALT/AST ratio	8	1.1: cut-off^b^	–	–
Triglycerides, mg/dL	–	0.007	–	0.953; log^c^
GGT, U/L	–	–	–	0.718; log^c^
Fasting insulin, mU/L	–	–	0.15	–

^a^If a subject satisfies the criteria, just adding the score of 2.

^b^If a subject has ALT/AST ratio ≥ 1.1, just adding the score of 1.

^c^The variable is used after log-transformation.

*HSI* hepatic steatosis index, *FSI* Framingham steatosis index, *LFS* *NAFLD liver fat score*, *FLI* fatty liver index, *AST* aspartate aminotransferase, *ALT* alanine aminotransferase, *GGT* gamma-glutamyl transferase.

### Statistics

All data in the KNHANES are presented as mean ± standard error for continuous variables and as frequency and percentage (%) for categorical variables. For continuous variables, we used a one-way analysis of variance to test for linear trends of the covariates after determining the mean values of each quartile group of the NAFLD index (e.g., HSI) as continuous variables. A chi-square test was used for categorical variables to compare differences among quartile groups. The comparison between two continuous variables (e.g., HSI vs. FLI) was performed using Pearson’s correlation coefficient (PCC).

Sample weights assigned to subjects were used to represent the whole Korean population. We used a linear regression model to identify the association between two continuous variables. In detail, we set muscle mass and each continuous form of an index for NAFLD as dependent and independent variables, respectively. For the relationship between the binary form of muscle status, including normal and LSMI (dependent variable) and each index for NAFLD (independent variable), we used a logistic regression model. For the multivariate model (i.e., linear and logistic regressions), we determined age, PA, current smoking, daily protein intake, hypertension, type 2 diabetes, dyslipidemia, cardiovascular disease (CVD), systolic BP, serum fasting glucose, total cholesterol, and serum creatinine as the confounding variables. All statistical analyses were conducted using R language (version 4.1.2). We set a *p*-value less than 0.05 as the significance level.

### Ethics approval

The present study was approved by the Institutional Review Board (IRB) of the K-CDC (IRB number: 2008-04EXP-01-C, 2009-01CON-03-2C, 2010-02CON-21-C, 2011-02CON-06-C).

### Consent to participate (ethics)

Informed consent had been obtained from all participants in the KNHANES.

## Results

### Comparison of features and their coefficients among four NAFLD scoring models

Table 2 presents the components and weights (coefficients or parameters) of the four NAFLD scoring models. HSI^8^, FSI^9^, LFS^10^, and FLI^11^ included four, seven, five, and four features, respectively. Most coefficients except two variables (i.e., female in the FSI model and AST/ALT ratio in LFS) exhibited positive values. In LFS, because the absence or presence of metabolic syndrome was used for the model, information about obesity and hyperlipidemia was included. Interestingly, a different direction of the parameter for gender was shown between HSI and FSI. Moreover, HSI and FSI used the ALT/AST ratio, whereas LFS used the AST/ALT ratio.

**Figure 1:**
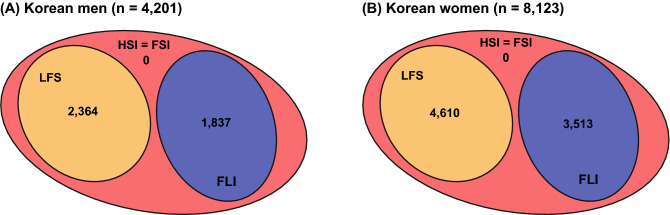
Comparison among scoring indices for fatty liver. Numeric values indicate the number of subjects with indices for fatty liver in men (**A**) and women (**B**).*HSI* hepatic steatosis index, *FSI* Framingham steatosis index, *LFS* NAFLD liver fat score, *FLI* fatty liver index.

The four models implemented different variables (Table 2). Therefore, the number of subjects with calculable results varied by the model (Fig. 1). Among the four scoring indices for NAFLD, the HSI and FSI provided the output of the model for all subjects (Fig. 1). No subjects had both LFS and FLI values (Fig. 1).

**Figure 2:**
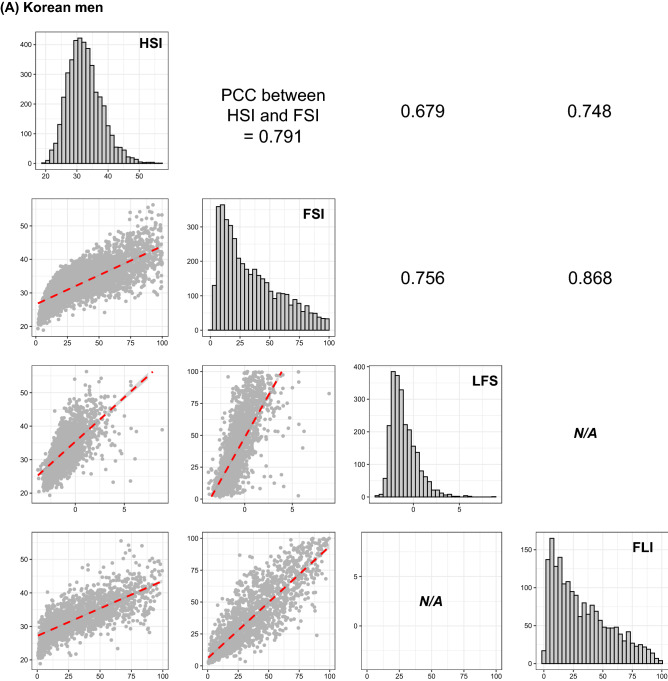

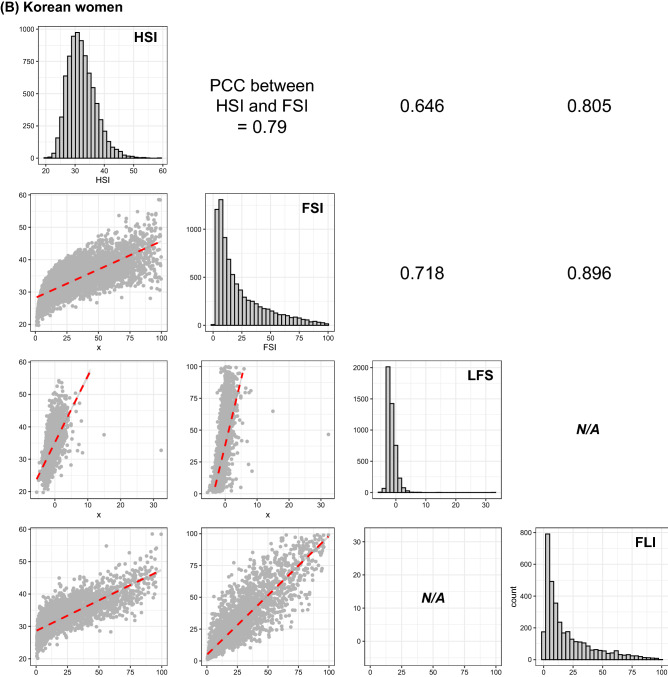
Correlation matrix among four indices for fatty liver in Korean men (**A**) and women (**B**). Distributions located in the diagonal are for each index for fatty liver. A scatter plot in the xth row and yth column indicates the correlation between xth index and yth index, and its slope is described by a red dotted line in the scatter plot and a value placed on the xth column and yth row. Comparison between LFS and FLI could not be performed because no sample had both data. Figures in the upper triangle matrix indicate PCC. *HSI* hepatic steatosis index, *FSI* Framingham steatosis index, *LFS* NAFLD liver fat score, *FLI* fatty liver index, *PCC* Pearson’s correlation coefficient.

**Figure 3:**
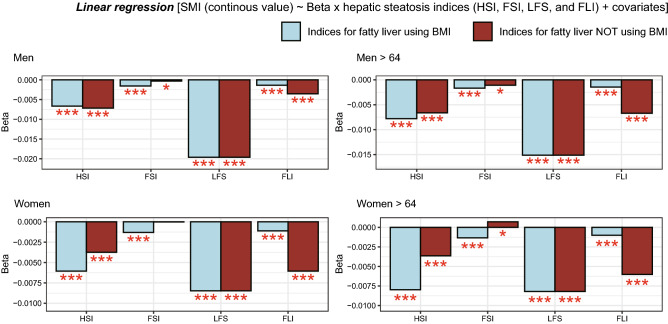
Relationships between SMI (ASM/BMI) and four hepatic steatosis indices. SMI and four indices are used as dependent and independent variables, respectively. Y-axes indicate beta-coefficients calculated by multivariate linear regression models including age, physical activity, current smoking, daily protein intake, hypertension, type 2 diabetes, dyslipidemia, CVD, systolic blood pressure, serum fasting glucose, total cholesterol, and serum creatinine as covariates. Brown bars indicate that the analysis was conducted using an index for fatty liver with a zero value of BMI coefficient. *SMI* skeletal muscle index, *ASM* appendicular skeletal muscle, *BMI* body mass index, *HSI* hepatic steatosis index, *FSI* Framingham steatosis index, *LFS* NAFLD liver fat score, *FLI* fatty liver index. *, **, *** indicate p-value < 0.05, < 0.01, < 0.001, respectively.

Figure 2 summarizes gender-specific correlations among all possible pairs in the four prediction models. The correlation between FSI and FLI exhibited the highest coefficients in both Korean men and women (Fig. 2). The HSI score was strongly correlated with that of FSI (PCC = 0.791 for men, 0.79 for women; *p*-value ≤ 0.001 in men and women; Fig. 2). Considering that the HSI was (1) based on the Korean population^8^, (2) could predict the status of NAFLD for all subjects in this study (Fig. 1), and (3) was highly correlated with the FSI that can also estimate the degree of NAFLD in all participants (Figs. 1, 2, and 3), we described the general characteristics of the present study according to the quartile of HSI score (Table 3).

**Table 3:** General characteristics of Korean men based on quartile of HSI level.

	Q1 (< 28.8)	Q2 (28.8–31.9)	Q3 (32.0–35.6)	Q4 (≥ 35.7)	*P* for trend
N	1050	1050	1050	1051	
HSI	26.3 ± 0.06	30.4 ± 0.03	33.8 ± 0.03	39.8 ± 0.11	< 0.001
FSI	6.2 ± 0.17	12.8 ± 0.37	18.3 ± 0.44	29.5 ± 0.63	< 0.001
LFS	−2.1 ± 0.03	−1.5 ± 0.04	−0.9 ± 0.04	0.3 ± 0.06	< 0.001
FLI	10.9 ± 0.47	24 ± 0.76	37.9 ± 0.84	58 ± 0.99	< 0.001
Age, years	53.4 ± 0.58	52 ± 0.53	51.4 ± 0.48	46.6 ± 0.45	< 0.001
ASM, kg	19.6 ± 0.09	21.5 ± 0.09	22.3 ± 0.09	24 ± 0.1	< 0.001
ASM/BMI, kg/kg/m^2^	0.9548 ± 0.0058	0.9212 ± 0.0056	0.8913 ± 0.0047	0.8852 ± 0.005	< 0.001
LSMI, n	92 (8.8)	116 (11)	150 (14.3)	182 (17.3)	< 0.001
Physical activity, n	287 (27.3)	276 (26.3)	289 (27.5)	253 (24.1)	0.253
Smoking, n	625 (59.5)	624 (59.4)	562 (53.5)	661 (62.9)	< 0.001
Daily protein intake, g	75.2 ± 1.37	79.9 ± 1.22	82.8 ± 1.31	85 ± 1.51	< 0.001
Hypertension, n	287 (27.3)	371 (35.3)	412 (39.2)	468 (44.5)	< 0.001
Type 2 diabetes, n	41 (3.9)	85 (8.1)	148 (14.1)	237 (22.5)	< 0.001
Dyslipidemia, n	45 (4.3)	78 (7.4)	112 (10.7)	142 (13.5)	< 0.001
CVD, n	43 (4.1)	55 (5.2)	56 (5.3)	62 (5.9)	0.297
Obesity, n	2 (0.2)	109 (10.4)	479 (45.6)	848 (80.7)	< 0.001
BMI, kg/m^2^	20.4 ± 0.05	23.1 ± 0.05	24.8 ± 0.05	27.1 ± 0.08	< 0.001
WC, cm	75.1 ± 0.2	82.6 ± 0.18	86.6 ± 0.18	92.1 ± 0.24	< 0.001
Systolic BP, mmHg	119.4 ± 0.54	121.5 ± 0.52	122.9 ± 0.47	122.9 ± 0.45	< 0.001
Diastolic BP, mmHg	75.2 ± 0.31	77.4 ± 0.31	79.8 ± 0.31	81.8 ± 0.32	< 0.001
Fasting glucose, mg/dL	93 ± 0.54	96.7 ± 0.57	101.3 ± 0.76	108 ± 1.02	< 0.001
Fasting insulin, mU/L	7.3 ± 0.12	8.9 ± 0.18	9.4 ± 0.15	12.5 ± 0.23	< 0.001
Total cholesterol, mg/dL	177.3 ± 0.98	182.9 ± 1.05	189 ± 1.07	195 ± 1.14	< 0.001
Triglycerides, mg/dL	98.9 ± 1.72	134.5 ± 2.74	153.1 ± 2.79	190 ± 3.86	< 0.001
HDL cholesterol, mg/dL	49.2 ± 0.34	44.8 ± 0.31	42.7 ± 0.28	40.1 ± 0.25	< 0.001
AST, U/L	22 ± 0.38	21.5 ± 0.25	22.7 ± 0.25	27.2 ± 0.37	< 0.001
ALT, U/L	15.7 ± 0.22	19 ± 0.23	24.6 ± 0.3	42.9 ± 0.81	< 0.001
GGT, U/L	30.3 ± 3.74	31.4 ± 1.9	37.3 ± 1.53	49.7 ± 1.64	< 0.001
Serum creatinine, mg/dL	0.9545 ± 0.0109	0.972 ± 0.0085	0.9816 ± 0.0082	0.9756 ± 0.0076	< 0.001

*HSI* hepatic steatosis index, *FSI* Framingham steatosis index, *LFS* NAFLD liver fat score, *FLI* fatty liver index, *ASM* appendicular skeletal muscle, *BMI* body mass index, *LSMI* low skeletal muscle index, *CVD* cardiovascular disease, *WC* waist circumference, *BP* blood pressure, *HDL* high-density lipoprotein, *AST* aspartate aminotransferase, *ALT* alanine aminotransferase, *GGT* gamma-glutamyl transferase.

### Gender-specific general characteristics according to the quartile of HSI

Table 3 presents general characteristics in Korean men according to the quartile of HSI levels. In detail, subjects were arranged in ascending order of HSI. Then, three points equally dividing the data by 25% were calculated. Based on the three cut-offs, all Korean men were categorized into four groups. As a result, the HSI quartiles were categorized as follows: Q1: < 28.8, Q2: 28.8–31.9, Q3: 32.0–35.6, Q4: ≥ 35.7. From the Q1 to Q4 HSI groups, the following characteristics exhibited stepwise increases: ASM, BMI, WC, systolic and diastolic BP, daily protein intake, fasting serum glucose and insulin, total cholesterol, triglycerides, AST, ALT, GGT, and serum creatinine and also higher rates of LSMI, hypertension, type 2 diabetes, and obesity (Table 3). The two variables, PA and CVD, had a non-significant relationship with HSI level.

Table 4 showed general characteristics in women based on the HSI quartile, categorized as follows: Q1: < 28.9, Q2: 28.9–31.7, Q3: 31.8–35.0, Q4: ≥ 35.1. Most of the trends were similar to those of the men, but there were differences in age, PA, and daily protein intake (Table 4).

**Table 4:** General characteristics of Korean women based on quartile of HSI level.

	Q1 (< 28.9)	Q2 (28.9–31.7)	Q3 (31.8–35.0)	Q4 (≥ 35.1)	*P* for trend
N	2031	2030	2031	2031	
HSI	26.9 ± 0.03	30.3 ± 0.02	33.3 ± 0.02	38.7 ± 0.07	< 0.001
FSI	3.4 ± 0.08	6.9 ± 0.15	13 ± 0.26	25.9 ± 0.41	< 0.001
LFS	−2.4 ± 0.02	−2 ± 0.02	−1.3 ± 0.04	−0.04 ± 0.04	< 0.001
FLI	4.2 ± 0.14	10.1 ± 0.27	20.3 ± 0.47	44.7 ± 0.73	< 0.001
Age, years	43.1 ± 0.37	49 ± 0.34	53.6 ± 0.32	54.5 ± 0.31	< 0.001
ASM, kg	13.3 ± 0.04	14.1 ± 0.04	14.6 ± 0.04	15.7 ± 0.05	< 0.001
ASM/BMI, kg/kg/m^2^	0.672 ± 0.0029	0.6236 ± 0.0026	0.5968 ± 0.0026	0.5671 ± 0.0025	< 0.001
LSMI, n	41 (2)	112 (5.5)	256 (12.6)	408 (20.1)	< 0.001
Physical activity, n	373 (18.4)	443 (21.8)	484 (23.8)	502 (24.7)	< 0.001
Smoking, n	167 (8.2)	140 (6.9)	132 (6.5)	122 (6)	0.036
Daily protein intake,	60 ± 0.66	58.6 ± 0.63	55.7 ± 0.63	55.4 ± 0.59	< 0.001
Hypertension, n	259 (12.8)	480 (23.6)	700 (34.5)	991 (48.8)	< 0.001
Type 2 diabetes, n	16 (0.8)	68 (3.3)	184 (9.1)	472 (23.2)	< 0.001
Dyslipidemia, n	73 (3.6)	145 (7.1)	257 (12.7)	403 (19.8)	< 0.001
CVD, n	26 (1.3)	33 (1.6)	89 (4.4)	86 (4.2)	< 0.001
Obesity, n	6 (0.3)	28 (1.4)	631 (31.1)	1695 (83.5)	< 0.001
BMI, kg/m^2^	19.7 ± 0.03	22.2 ± 0.03	24.2 ± 0.04	27.4 ± 0.06	< 0.001
WC, cm	69 ± 0.12	75.5 ± 0.13	81.4 ± 0.14	89.1 ± 0.18	< 0.001
Systolic BP, mmHg	109.9 ± 0.36	115.6 ± 0.4	120.5 ± 0.41	125 ± 0.4	< 0.001
Diastolic BP, mmHg	70.7 ± 0.2	73.5 ± 0.22	75.6 ± 0.23	78.4 ± 0.23	< 0.001
Fasting glucose, mg/dL	88.9 ± 0.18	92.3 ± 0.37	97.6 ± 0.48	106.3 ± 0.65	< 0.001
Fasting insulin, mU/L	7.9 ± 0.09	8.4 ± 0.08	10 ± 0.23	12.7 ± 0.19	< 0.001
TC, mg/dL	176.8 ± 0.67	187.5 ± 0.74	195.3 ± 0.84	198.5 ± 0.84	< 0.001
Triglycerides, mg/dL	82.1 ± 1.02	102.6 ± 1.45	127.2 ± 1.89	151 ± 2	< 0.001
HDL cholesterol, mg/dL	53.6 ± 0.24	51.2 ± 0.25	48.4 ± 0.24	45.9 ± 0.22	< 0.001
AST, U/L	18.2 ± 0.14	19.1 ± 0.13	20.3 ± 0.15	23.3 ± 0.24	< 0.001
ALT, U/L	11.7 ± 0.09	14.4 ± 0.13	17.6 ± 0.17	26.6 ± 0.43	< 0.001
GGT, U/L	14.9 ± 0.35	17.3 ± 0.39	20.2 ± 0.47	31 ± 1.19	< 0.001
Serum creatinine, mg/dL	0.6969 ± 0.0033	0.7041 ± 0.0036	0.715 ± 0.0042	0.7164 ± 0.0046	< 0.001

*HSI* hepatic steatosis index, *FSI* Framingham steatosis index, *LFS* NAFLD liver fat score, *FLI* fatty liver index, *ASM* appendicular skeletal muscle, *BMI* body mass index, *LSMI* low skeletal muscle index, *CVD* cardiovascular disease, *WC* waist circumference, *BP* blood pressure, *HDL* high-density lipoprotein, *AST* aspartate aminotransferase, *ALT* alanine aminotransferase, *GGT* gamma-glutamyl transferase.

### Relationships between the four hepatic steatosis indices and muscle mass

The relationships between fatty liver indices and SMI (ASM/BMI) were analyzed using multivariate linear regression and a weighted population. In both men and women, all four indices for fatty liver exhibited a negative relationship with the SMI in all age groups and over 65 years old (light-blue bars in Fig. 3). Since three of the four indices for fatty liver and SMI contain BMI as a predictor in common, significant correlations are expected between them. In other words, more evidence is required to conclude that muscle mass is related to fatty liver. Therefore, we calculated indices for fatty liver after removing the BMI effect. Two methods could be used to remove the effect of BMI, including altering the coefficient of BMI to zero in the four NAFLD models or changing BMI levels to zero in the data. Among them, we changed the coefficients for BMI in each index to zero and then conducted associational analyses between each index without the BMI effect and SMI (ASM/BMI). For the HSI and FLI, the negative association with SMI remained after removing the BMI effect in both men and women (brown bars in Fig. 3). The LFS does not include BMI as a component. Thereby, there is no change after removing the BMI effect. In the analyses for Korean men and women aged 65 or over, most results, except the association between FSI and SMI in Korean women, were consistent with those of all subjects aged ≥ 20 (Fig. 3).

### Relationships between the four indices of fatty liver and LSMI

**Figure 4:**
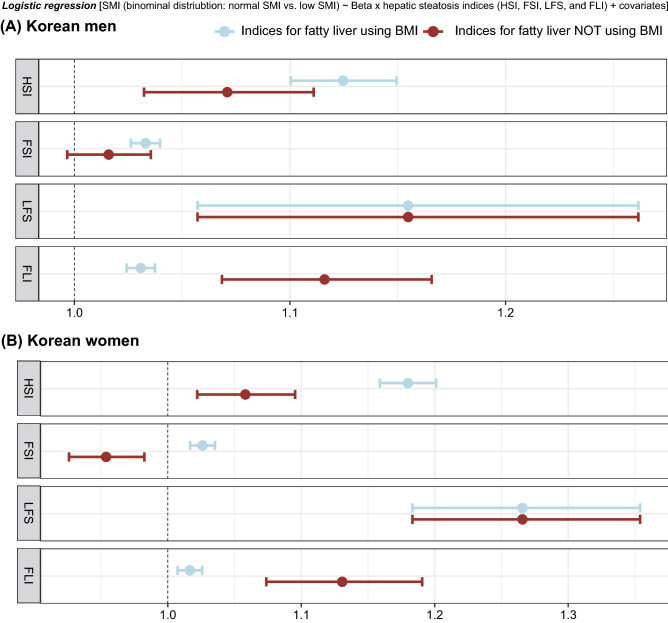
Relationships between LSMI and four hepatic steatosis indices. LSMI status (presence vs. absence) and four indices in Korean men (**A**) and women (**B**) were used as dependent and independent variables, respectively. X-axes indicate odds ratio and 95% confidence interval calculated by multivariate logistic regression models, which include age, physical activity, current smoking, daily protein intake, hypertension, type 2 diabetes, dyslipidemia, CVD, systolic blood pressure, serum fasting glucose, total cholesterol, and serum creatinine as covariates. Brown points and lines indicate that the analysis was conducted using indices for fatty liver with a zero value of BMI coefficient. *LSMI* low skeletal muscle index, *BMI* body mass index, *HSI* hepatic steatosis index, *FSI* Framingham steatosis index, *LFS* NAFLD liver fat score, *FLI* fatty liver index.

Using multivariate logistic regression, we measured the degree of the relationship between fatty liver indices for the binary form of LSMI (normal vs. LSMI). Similar to the above linear regression analyses, a weighted population was used. As a result, the increased levels of the four indices for fatty liver exhibited a significant association with an increased risk of LSMI (blue points in Fig. 4). After removing the BMI effect with the three indices (i.e., HSI, FSI, and FLI), the associations with the ratio LSMI in two of them (HSI and FLI) remained significant in both men and women (brown points in Fig. 4).

## Discussion

We reviewed four models estimating fatty liver by comparing the components they included and how the predictors of each model contribute to the final result (e.g., positive or negative coefficient value for a predictor). Then, we compared the statistical power and direction of the relationship between each model with LSMI. We found that all fatty liver models exhibited a relationship between high scores and a high prevalence of LSMI. However, the above results might be biased since fatty liver models and LSMI included BMI information. Therefore, we removed the effect of BMI in each fatty liver model and re-analyzed the relationship between fatty liver and sarcopenia. Most fatty liver models exhibited a robust and positive relationship with the increased prevalence of LSMI in men and women.

LSMI also referred to as sarcopenia, has been considered a risk factor for NAFLD^28^. In a Korean study examining subjects who visited the Health Promotion Center at Samsung Medical Center, high muscle mass measured by bioelectrical impedance analysis (BIA) was significantly related to low incident NAFLD diagnosed by HSI^8^. Lee et al.^16^ analyzed the KoGES dataset, a nationwide longitudinal study, demonstrating that LSMI determined by BIA exhibited an increased ratio of new-onset NAFLD defined by LFS. In cross-sectional studies, most analyses were performed after determining muscle mass or strength as an independent variable, and NAFLD was determined by several estimating models as a dependent variable^6,7,29^. However, we have an opposing view on this point because the prevalence of NAFLD at a young age was reported as 22% (95% CI: 15.38–31.52)^30^, and that of sarcopenia in the population aged > 60 was 5 to 13%^31–33^. We suggest that the relationship between NAFLD and LSMI might occur as a dual etiology concept, web-like reaction, or bidirectional association. Since NAFLD tends to occur at a younger age than sarcopenia, it is rational to consider NAFLD as a prerequisite status or risk factor for sarcopenia. Therefore, in this study, several indices for fatty liver were arranged as independent variables and LSMI as a dependent variable.

BMI or its components, including weight and height, were used as predictors for HSI^8^, FSI^9^, and FLI^11^. They were also used to define LSMI or sarcopenia, yielding the biased and definitive association between NAFLD and LSMI. To overcome this limitation, several studies conducted adjustments for obesity status (Table 1)^7,29^. In our study, we performed an associational analysis between NAFLD and LSMI after removing the effect of BMI. The coefficient for BMI was changed to zero, resulting in decreased beta-coefficients between HSI and LSMI and a non-significant or reverse association between FSI and LSMI (Fig. 4). In case of FLI, the beta-coefficient for the presence of LSMI increased after removing the effect of BMI. An explanation for this could be that the FLI includes WC, which highly correlates with BMI as a predictor (Table 2).

The Danish National Registry of Patients analyzed about 1,800 patients diagnosed with NAFLD and found that CVD-related mortality increased in the NAFLD group compared with the general population^34^. However, in our study, a significant association between NAFLD and CVD was shown in only Korean women. A randomized, controlled trial (RCT) found that 16 weeks of exercise training group (12 subjects with NAFLD) showed insignificant improvement in hepatic lipoprotein kinetics (hepatic lipoprotein secretion), compared with the control group (n = 6)^35^. Zhang et al.^36^ conducted exercise intervention for subjects with central obesity and NAFLD, resulting in vigorous and moderate exercise intensity significantly reduced intrahepatic triglyceride content. In our study, an insignificant relationship between NAFLD and PA was shown in Korean men, and a significant but inconsistent findings with those obtained from the study by Zhang et al.^36^ were resulted in Korean women. These non-converging findings might be resulted because our study evaluated PA by integrating several criteria obtained from self-response data^6,16,17^.

Several plausible mechanisms for the significant association between NAFLD and sarcopenia are suggested, including chronic low-grade inflammation, oxidative stress, mitochondrial dysfunction, and insulin resistance^37,38^. Tarantino et al.^39^ reviewed several mechanisms for the pathogenesis of NAFLD and pinpointed chronic inflammation as the key mechanism of insulin resistance as well as the progression of NAFLD. Chung et al.^40^ demonstrated that the chronic low-grade inflammation evaluated by blood WBC count was related to a high risk of sarcopenia. Insulin resistance has been considered an integral component of the NAFLD mechanism^41^. Not surprisingly, patients with NAFLD exhibit an increased risk for new-onset diabetes^42^. Moreover, sarcopenia shares close pathogenesis mechanisms with biological alterations triggered by insulin resistance^43^. Ours is not a functional study but a clinical and observational study. Therefore, we cannot provide data on the mechanism between NAFLD and sarcopenia. However, the above findings suggest that a critical mechanism for the association between NAFLD and LSMI involves mitochondrial function and cell respiration.

## Limitations and conclusion

There were several limitations in this study. First, as diagnostic tools for fatty liver, imaging data such as US, CT scan, and magnetic resonance imaging were not available. Albeit the attempt to remove the BMI effect, we could not complete its trace due to the BMI-related factors. For example, the FLI^11^ included WC, which is strongly correlated with BMI level, and the FSI^9^ consisted of a MetS diagnosis for which the central obesity component is required. Therefore, a future study including liver imaging data obtained from the above tools is required to confirm our hypothesis. Moreover, histological findings obtained from the liver biopsy are required to measure the severity of NAFLD or distinguish the presence of steatohepatitis. Second, we could not reveal the association between NAFLD and other sarcopenic criteria due to a lack of data on muscle strength and function. A study that includes subjects with muscle mass, strength, and functional data are required to demonstrate the generalized association between NAFLD and sarcopenia. Lastly, the causal relationship between NAFLD and sarcopenia cannot be determined because of the cross-sectional research. Lee et al.^16^ analyzed a longitudinal cohort and demonstrated that LSMI defined by BIA is a significant risk factor for incident LSMI. If a longitudinal study is designed to include muscle mass obtained from DEXA and muscle strength, it could confirm this hypothesis.

We reviewed four models predicting hepatic steatosis and found that FSI-FLI and HSI-FLI exhibited the best and second-best correlations among all possible pairs. Then, we demonstrated that the four models were significantly associated with LSMI. Moreover, after removing the BMI effect, the high values obtained from HSI and FLI were robust predictors for increased risk of LSMI.

## Data Availability

All data is publicly available (https://knhanes.kdca.go.kr/knhanes/eng/index.do).
